# Coxa Vara

**Published:** 1902-08-30

**Authors:** A. H. Tubby

**Affiliations:** Surgeon to Westminster, the Evelina, and National Orthopædic Hospitals.


### Hospital Clinics and Medical Progress.

### COXA VARA.

By A. H. Tubby, M.S., F.R.C.S., Surgeon to Westminster, the Evelina, and National Orthopædic Hospitals.

Coxa vara is a deformity of the upper part of the femur which results in depression of the head of the femur and an alteration of the axis of the neck. There are various causes from which this deformity may arise, but they may be briefly summed up thus :—

If for any reason, such as rickets, osteomalacia, acute osteitis, or tubercular osteitis, the upper part of the femur undergoes softening, the effect of the weight of the body is to alter the shape of the upper third of the thigh bone. As a rule the neck of the femur yields most, and the head, from being inclined at a very obtuse angle, becomes less obtuse, then horizontal, then is placed at an acute angle, and finally may become merely a globular mass, sessile on the femur, and one inch or so below the level of the top of the great trochanter. In rare cases, however, it is not so much the neck which yields as it is that the upper part of the shaft of the femur bends outwards just below the trochanters. But such cases are exceptional. There are certain cases in which a somewhat similar condition arises from injury. In these either fracture of the neck of the femur or separation of the epiphysis in that situation occurs, and, if the union be not perfect, the angle of inclination of the head and neck to the shaft is lessened. These are instances of so-called traumatic coxa vara.

But to return more particularly to cases of spontaneous depression of the neck and head of the femur. We think that the term coxa vara should be limited to those examples where the process is due to a slow softening of bone occurring in connection with rhachitis adolescentium. Coxa vara is therefore a disease of adolescence and rarely of adult life. It is more often unilateral than bilateral.

The symptoms to which it gives rise are the following : Some pain and disability in walking, accompanied by a variable amount of shortening of the limb. The trochanter is often situated above Nélaton's line, and as the inclination of the shaft of the femur becomes more definitely downwards and inwards there is lessening of the power of abducting the limb, and subsequently considerable contraction of the adductor muscles takes place. As a rule there is no limitation of flexion except under the circumstances to be narrated below. Therefore in the case of an adolescent who presents himself with a shortened limb, inclination of the lower end of the femur inwards, free movements in all directions except abduction and no thickening about the hip joint ; and if it be felt that the head of the femur is in the acetabulum, the diagnosis of coxa vara is fairly evident. The peculiar deformity arising from it may very often be best seen if the limbs are flexed ; and if the case be one of bilateral coxa vara the thighs will become much adducted and even cross one another so as to give rise to what is known as "scissor-leg" deformity.

Mention has been made above of flexion at the hip joint being sometimes interfered with. The explanation of this is as follows :—The head of the femur does not sink directly downwards, nor is the bending of the neck in a truly vertical plane, but sometimes the neck undergoes, as well as a downward deformity, an antero-posterior bending—that is, the shape of the neck of the femur is more horizontal and it is convex forwards. It is this anterior convexity which limits flexion. In still more rare cases the neck of the femur becomes convex backwards and extension is interfered with.

The diagnosis should be made from coxitis, especially from tubercular coxitis, and from congenital dislocation. In tubercular coxitis, as a rule, there is no shortening at the first onset. The limb is abducted and not adducted, loss of complete flexion is very early seen, and later we have thickening of the head and neck of the femur and the great trochanter, and pain of an intermittent character. The child's general health also suffers. In congenital dislocation of the head of the thigh bone we have the history that it was first noticed at the time of walking. We also observe that the head of the bone is not in its proper position, and, further, that it can be moved in a vertical direction by pressure in an upward direction on the thigh bone as the patient lies recumbent. The best diagnostic test in doubtful cases, however, is a Roentgen ray photograph, and it is curious to note that many cases which have been thought to be coxa vara have proved on examination by this method to be cases of partial dislocation of the femur.

The treatment of coxa vara depends largely upon the degree it has assumed. In slight cases, that is those in which the shortening is limited to half an inch, complete rest in bed, thereby removing the weight of the body from the weakened neck of the femur, is the first essential. If with this rest there be combined weight-extension, with the leg in an abducted position at an angle of 45°, the deformity is arrested, and in some cases the shortening disappears. It may be asked how long will this take ? At least several months, and the question arises : When shall weight extension be removed and the patient allowed to get about ? If the shortening has been arrested and if no increase in length of the limb has been obtained during the last two months of treatment it may be taken that the softening process in the neck of the femur has ceased and that the bone has become hard again, and therefore the patient may be allowed to get up. But care should be exercised that he does not throw too much strain upon the affected part, and he should have a cork sole to compensate for the shortcoming.

In those instances where the shortening is more than half an inch, where there is much adduction of the femur, contraction of the adductor muscles and loss of flexion, the best form of treatment is a femoral osteotomy. Various surgeons select various positions. In my opinion the best line of division of the bone is an oblique intertrochanteric one. This should be combined with section of the contracted adductor tendons, and after the operation the limb should be put at an angle of 45° and kept there for six weeks. The results of this operation are quite satisfactory.

